# Communication about diagnosis and prognosis—A population‐based survey among bereaved parents in pediatric oncology

**DOI:** 10.1002/pon.6058

**Published:** 2022-11-04

**Authors:** Cecilia Bartholdson, Ulrika Kreicbergs, Josefin Sveen, Malin Lövgren, Lilian Pohlkamp

**Affiliations:** ^1^ Childhood Cancer Research Unit Department of Women's & Children's Health Karolinska Institutet Stockholm Sweden; ^2^ Paediatric Neurology and Musculoskeletal Disorders and Homecare Children's and Women's Healthcare Karolinska University Hospital Stockholm Sweden; ^3^ Department of Health Care Sciences Palliative Research Centre Marie Cederschiöld University College (formerly Ersta Sköndal Bräcke University College) Stockholm Sweden; ^4^ Department of Women's and Children's Health Uppsala University Uppsala Sweden; ^5^ Advanced Pediatric Home Care Karolinska University Hospital Stockholm Sweden

**Keywords:** cancer, children, communication, diagnosis, oncology, parents, prognosis, psycho‐oncology

## Abstract

**Introduction:**

When a child is diagnosed with cancer, the whole family is affected, and parents struggle to grasp challenging information regarding diagnosis and prognosis. Most parents and children want honest communication and openness, yet this remains a complex and challenging task for healthcare professionals.

**Objectives:**

To describe bereaved mothers' and fathers' reports of communication of their child's cancer diagnosis and when the illness became incurable.

**Methods:**

Data from a Swedish population‐based survey conducted in 2016, including 135 mothers and 97 fathers who had lost a child to cancer 1–5 years earlier, were studied regarding the parents' reports of communication about their child's illness.

**Results:**

A vast majority of parents wants information when their child's illness becomes incurable, and this need is generally met. However, fathers to a lesser extent than mothers, reported that they were informed about it. According to parents' reports 87% of children received diagnostic information and 44% of the children received prognostic information.

**Conclusion:**

A vast majority of both mothers and fathers would like to know when their child's illness becomes incurable, yet it remains unknown to what extent they want their child to be informed.

## 1 INTRODUCTION

When a child is diagnosed with cancer, the whole family is affected and many parents suffer physical, spiritual, psychological and social struggles when caring for their child with cancer, regardless if the cancer is expected to be curable.^1^, ^2^ Parents often display strong emotional reactions when receiving bad news about their child's illness and have described feelings such as vulnerability, powerlessness, anger, anxiousness and fear related to the content in the news.^3^ It is also known that the news itself is challenging for parents to grasp and assimilate. One study showed that close to one out of 10 parents stated that they could not grasp the information about their child's incurable cancer at all, and one fifth only to some extent.^4^

End‐of‐life communication with families is experienced as a difficult and challenging task by healthcare professionals, many paediatric oncologists feel discomfort and agony about being trained to cure, but being forced to break bad news to parents of children with cancer.^5^ Although parents often involve their children and encourage them to be participants in their care,^6^ sometimes parents do not want clinicians to talk about death with the dying child. This has been experienced as another reason for stress for physicians and an ethical issue in paediatric oncology,^7^ it has also been found to be morally distressing by healthcare professionals.^8^

In a recent study where barriers and facilitators for breaking bad news were identified,^9^ the authors concluded that it was important to talk to parents in a timely manner about the fact that their child might not survive, even if the prognosis was uncertain, and that professionals needed to improve how they communicated bad news.^9^, ^10^ Healthcare professionals can support parents through empathetic communication about their child's diagnosis, treatment and prognosis, repeated on several occasions to prevent overload of information and create continuity.^11^, ^12^

It is important to gain extended knowledge about parents' experiences of communication with healthcare professionals when their child receives a cancer diagnosis and when their child's illness becomes incurable. Such knowledge may facilitate important family communication and communication between family and professionals. Furthermore, new methods for communication can be developed and evaluated when empirical knowledge, based on parents' experiences, is reached. Therefore, the aim of this study was to describe bereaved mothers' and fathers' reports of communication of their child's cancer diagnosis and when the illness became incurable.

## 2 METHODS

### 2.1 Study design

A population‐based nationwide survey was conducted in 2016 where bereaved parents' psychological health and reports of healthcare experience were examined.

### 2.2 Study participants

Study participants were parents in Sweden whose children had died due to cancer 1–5 years earlier. The participants were identified through the Swedish Childhood Cancer Registry (diagnosis age 0–16 years), which is a national database including children who have received a childhood cancer diagnosis, combined with the Cause of Death Registry, reporting children who had died from their childhood cancer diagnosis (0–24 years), and the Swedish Population Register at the Swedish Tax Agency.

Inclusion criteria were parents living in Sweden who had sufficient knowledge of Swedish to be able to answer the study‐specific questionnaire and who had lost a child or young adult (age 0–24 years) to childhood cancer between 2010 and 2015.

### 2.3 Data collection

Eligible parents (*n* = 530) were invited through an information letter describing the purpose of the study, sent by the research group. When 2 weeks had passed, each parent of each child was contacted by telephone with a query of consent to participate. Parents who themselves had contacted the researchers within the 2 weeks were not contacted. In cases where parents did not have a listed telephone number, a request was sent by post requesting them to contact the research group. A questionnaire with a prepaid return envelope was sent to parents who consented to participate. One reminder call to those who had not responded to the survey was conducted after a few weeks, attempting not to put any pressure on the parents. Out of 373 parents who consented to participate, 232 returned the questionnaire. Demographics of parents are shown in Table 1 and children's demographics are shown in Table 2.

**TABLE 1:** Characteristics of participating parents at time of the study^a^

Characteristics of parents at time of the study (Nov 2016)		
	Mean (SD)	Median (range)
Parent age at the time of the study (*n* = 232)	46 (8.27)	46 (24–67)
Parent age at child death (*n* = 232)	42 (8.11)	41 (20–62)
Years since loss (*n* = 232)	4 (1.44)	4 (1–6)
Number of children at the child's diagnose (*n* = 229)	2.3 (1.09)	2 (1–8)
Parent sex (*n* = 232)		*n* (%)
Female		135 (58)
Male		97 (42)

	Mothers *n* (%)	Fathers *n* (%)	Total *n* (%)
Marital status (*n* = 231)			
Married	120 (89)	87 (90)	207 (89)
Living apart (but as a couple)	2 (2)	2 (2)	4 (2)
Single	11 (8)	8 (8)	19 (8)
Total by sex	133	97	231
Main occupation at the child's diagnose (*n* = 229)			
Employed	98 (73)	90 (93)	188 (81)
Studying	2 (2)	2 (2)	4 (2)
Parental leave	22 (16)	1 (1)	23 (10)
Unemployed	4 (3)	1 (1)	5 (2)
Sick leave	4 (3)	1 (1)	5 (2)
House ‐wife/husband	2 (2)	2 (2)	4 (2)

^a^ Figures in brackets in the result columns are percentages.

**TABLE 2:** Characteristics of the deceased children

Characteristics of the deceased children (*N* = 156)		
Type of paediatric cancer of the children (*n* = 152)		*n* (%)
Brain tumour		61 (39)
Leukaemia/Lymphoma		45 (29)
Sarcoma		20 (13)
Neuroblastoma		8 (5)
Other cancers		18 (12)
Child sex (*n* = 156)		*n* (%)
Female		69 (44)
Male		87 (56)
Ages and time of illness (years) (*n* = 156)	Mean (SD)	Median (range)
Child age at diagnosis	7.35 (5.3)	7 (0–16)
Child illness length	2.71 (3.6)	1 (0–21)
Child age at death	10.08 (6.6)	10 (0–24)
Children suffered from relapse. (*n* = 149)		*n* (%)
Yes, one time		44 (28)
Yes, several times		23 (15)
No, the illness never disappeared despite treatment		82 (53)
Children underwent stem cell transplantation. (*n* = 154)		*n* (%)
Yes, one time		31 (20)
Yes, several times		5 (3)
No		118 (76)

### 2.4 The survey

The survey covered sociodemographic characteristics and study‐specific questions about the parents' experiences of their child's illness and death, as well as parents' symptoms of psychological health and prolonged grief 1–5 years after the loss of their child using standardised instruments. The survey was validated through face‐to‐face interviews with cancer‐bereaved parents regarding relevance and clarity of the items. The items of the survey included in this study focused on the parents' reports of communication about diagnosis and prognosis between children, parents, and healthcare professionals (Tables 3 and 4). Study results on the bereaved parents' symptoms of psychological health and prolonged grief have been reported in other publications.^13^, ^14^, ^15^, ^16^

**TABLE 3:** Timeframes related to symptom development and diagnosis^a^

	Mothers *n* (%)	Fathers *n* (%)	Total *n* (%)
How long time from development of symptoms to contact with HC^b^ (*n* = 221)			
Couple of days	52 (39)	40 (41)	92 (40)
Couple of weeks	41 (30)	32 (33)	73 (32)
A month	26 (19)	11 (11)	37 (16)
Several months up to 1 year	8 (6)	8 (8)	16 (7)
One year or longer	1 (<1)	1 (1)	2 (1)
Total by sex	129	92	221
How long time from initial contact with HC until receiving diagnose (*n* = 223)			
Couple of days	55 (41)	40 (41)	95 (41)
Couple of weeks	29 (22)	22 (23)	51 (22)
A month	23 (17)	14 (14)	37 (16)
Several months up to 1 year	21 (16)	12 (12)	33 (14)
One year or longer	3 (2)	3 (3)	6 (3)
We never received a diagnose	0 (0)	1 (1)	1 (<1)
Total by sex	131	92	223

^a^ Figures in brackets in the result columns are percentages.

^b^ HC = Healthcare.

**TABLE 4:** Descriptions of how parents received information about their child's illness and perceptions of the situation when receiving ^a^

	Mothers *n* (%)	Fathers *n* (%)	Total *n* (%)
How did you get the information that your child had cancer? (*n* = 229)			
With my child and the other parent	68 (50)	48 (50)	116 (50)
With the other parent	25 (19)	19 (20)	44 (19)
With my child without the other parent	22 (16)	6 (6)	28 (12)
I was alone	9 (7)	10 (10)	19 (8)
Other ways	8 (6)	12 (12)	20 (9)
N/A I never got such information	0 (0)	1 (1)	1 (<1)
Total by sex	133	96	229
How did your child get the information that he/she had cancer? (*n* = 227)			
With me and the other parent	81 (60)	60 (62)	141 (61)
With me without the other parent	26 (19)	6 (6)	32 (14)
With the other parent without me	2 (2)	13 (13)	15 (7)
Without parents present	0 (0)	0 (0)	0 (0)
Other ways	4 (3)	4 (4)	8 (3)
N/A he/she never got such information	20 (15)	11 (11)	31 (13)
Total by sex	133	94	227
Who gave the information that your child had cancer? (*n* = 229)			
Physician at the healthcare centre	5 (4)	1 (1)	6 (3)
Physician at the hospital	81 (61)	55 (57)	136 (59)
Physician at a paediatric oncology unit	45 (34)	36 (38)	81 (35)
Other	2 (2)	4 (4)	6 (3)
Total by sex	133	96	229
How did you get the information that your child's illness was incurable? (*n* = 228)			
With my child and the other parent	31 (23)	26 (27)	57 (25)
With the other parent	61 (45)	40 (41)	101 (44)
With my child without the other parent	5 (4)	0 (0)	5 (2)
I was alone	13 (10)	8 (8)	21 (9)
Other ways	4 (3)	7 (7)	11 (5)
N/A I never got such information	19 (14)	14 (14)	33 (14)
Total by sex	133	95	228
How did your child get the information that his/her illness was incurable? (*n* = 227)			
With me and the other parent	36 (27)	29 (29)	65 (28)
With me without the other parent	10 (7)	1 (1)	11 (5)
With the other parent without me	1 (<1)	4 (4)	5 (2)
Without parents present	4 (3)	3 (3)	7 (3)
Other ways	4 (3)	5 (5)	9 (4)
N/A he/she never got such information	77 (57)	53 (55)	130 (56)
Total by sex	132	95	227
Do you believe that physicians should give information about when hope of cure ends? (*n* = 223)			
Yes	124 (92)	92 (95)	216 (93)
No	4 (3)	3 (3)	7 (3)
Total by sex	128	95	223
How long time before your child died did you get the information that the illness was incurable? (*n* = 226)^b^			
Within 24 h	11 (8)	10 (10)	21 (9)
A couple of days before	8 (6)	11 (11)	19 (8)
A week before	8 (6)	5 (5)	13 (6)
2–4 weeks before	17 (13)	17 (18)	34 (15)
1–3 months before	27 (20)	17 (18)	44 (19)
4–6 months before	15 (11)	12 (12)	27 (12)
7–11 months before	9 (7)	9 (9)	18 (8)
One year or longer before	18 (13)	6 (6)	24 (11)
N/A I never got such information	18 (13)	8 (8)	26 (11)
Total by sex	131	95	226
Was the information about your child's incurable cancer delivered in a respectful way? (*n* = 217)			
Completely agree	71 (53)	47 (49)	118 (51)
Mostly agree	27 (20)	23 (24)	50 (22)
Slightly agree	15 (11)	14 (14)	29 (13)
Completely disagree	12 (9)	8 (8)	20 (9)
Total by sex	125	92	217
Could you absorb the information that your child's illness was incurable? (*n* = 214)			
Completely agree	41 (30)	33 (34)	74 (32)
Mostly agree	43 (32)	32 (33)	75 (32)
Slightly agree	26 (19)	18 (19)	44 (19)
Completely disagree	15 (11)	6 (6)	21 (9)
Total by sex	125	89	214
Do you have trust in that healthcare did everything possible to cure your child? (*n* = 225)			
Completely agree	68 (50)	54 (56)	122 (53)
Mostly agree	36 (27)	25 (26)	61 (26)
Slightly agree	20 (15)	8 (8)	28 (12)
Completely disagree	7 (5)	7 (7)	14 (6)
Total by sex	131	94	225

^a^ Figures in brackets in the result columns are percentages.

^b^ There was a statistical difference between mothers and fathers, meaning that fathers to a lesser extent got the information that their child's illness was incurable (*p* = 0.048).

### 2.5 Analysis

Each questionnaire was assigned a number based on respondent ID. Questionnaires with less than 10% missing responses were considered acceptable. Data analysis was performed using the IBM SPSS Statistics for Windows, version 22 (IBM Corp.). Using an exploratory approach general characteristics of the deceased children and parents, as well as parents' responses to the items, were analysed using descriptive statistics analysis (frequency, percentage, mean, standard deviation [SD], median, range). The Wilcoxon signed‐rank test, a non‐parametric paired *t*‐test, was used to test differences between mothers and fathers in relation to marital status and occupation. The significance level (*p*‐value) was set to < 0.05.

### 2.6 Ethical considerations

Parents received an information letter that described the possibility to participate in a nationwide survey and about the voluntary nature of participation. Once started it was possible to cancel participation without explaining why. Contact information to responsible researchers was also provided. Consent to participate was gathered by telephone, as described above. The study was approved by the Regional Ethical Review Board in Stockholm, Sweden (No: 2015/2183‐31/5).

## 3 RESULTS

This study includes responses from 232 parents. The number was obtained after excluding the following from the initial eligible parents in registries (*N* = 530): 18 bereaved parents did not meet the criteria; 76 bereaved parents could not be contacted (no e‐mail/telephone number); 63 bereaved parents declined to participate. Thereafter, 373 consented to participate but 141 did not return the questionnaire. Characteristics of parents included: age at time of study, age when their child died, years since loss, number of children at the time of the child's diagnosis, sex, marital status and main occupation (Table 1). Characteristics of the deceased children included: type of paediatric cancer, sex, age, duration of illness, incidence of relapse and stem cell transplantation (Table 2). Background information including parents' reports on the time from symptom onset to contact with healthcare and the time from contact with healthcare until diagnosis are described in Table 3. It is important to note that almost three quarters of the parents reported that they contacted healthcare within a couple of days to a couple of weeks since development of symptoms. Two thirds of the parents report that the child received a diagnose within a couple of days to a couple of weeks since contact with healthcare.

### 3.1 Communication about the child's cancer

Half of the parents (50%) reported that they received the information that their child had cancer, together with the child and the other parent (Table 4). In 12% of the cases, one of the parents was alone with their child when receiving the information of a cancer diagnosis. Both mothers and fathers reported that mothers were more often alone with the child when receiving the diagnosis, than fathers. When parents answered the question if their child got the information that he/she had cancer, 13% stated that their child never received such information. These 13% represent 22 unique children. There was an equal distribution across child sexes within in this group (female, *n* = 10; male, *n* = 12). Mean age of those children was 2.6 years (SD = 4.3), range (0–15).

Almost all (93%) mothers and fathers in the study stated that physicians should communicate openly when hope for cure ends. When receiving the information that their child's cancer was incurable, 44% of the parents report they were together with the other parent, but without the child present, while 25% of the parents reported that their child was present. Fourteen percent of the parents reported that they never got the information that their child's cancer was incurable and 3% of the parents reported that their child was alone when receiving information about the illness being incurable. These 3% represent 4 unique children and analysis revealed that their mean age was 7.3 years (range 1–15 years) when the children were diagnosed, and the mean age was 18.0 years (range 16–22 years) when they died. No data are available on the children's ages when receiving prognostic information. Furthermore, in more than half of the cases (56%), parents reported that their children never got the information that their cancer was incurable. Among them (87 unique children) the gender distribution was almost equal, 40 girls and 47 boys and the mean age was 6.5 years (SD = 5.1), range (0–16). Thus, the number of children present when bad news was communicated, decreased with increasing degree of severity of their illness. Meaning that fewer children attended when information about their cancer being incurable was given compared to when information about diagnosis was given. Mothers reported that they were alone with the child more often than fathers (10 vs. 1%) when their child got the information that the cancer was incurable.

Parents' responses to the question on how long before their child died, they had received information that the cancer was incurable, ranged from ‘within 24 h’ to ‘1 year or longer before’. When asked whether the communication about the child's incurable cancer was delivered in a respectful way, half of the parents (51%) completely agreed, whereas 22% mostly agreed. Nine percent completely disagreed. Most parents (64%) agreed that they could in some way grasp the information that their child's cancer was incurable. Some (9%) completely disagreed that they could grasp the information. Half of the mothers (50%) and just above half of the fathers (56%) reported that they completely agreed that they believed that healthcare professionals did everything possible to cure their child, while 6% of the parents completely disagreed. More detailed data are shown in Table 4.

### 3.2 Possible influence of demographic factors

No significant differences were found in reports between parents who had a child with a brain tumour, which was the most common diagnosis, and parents who had a child with other diagnoses. When analysing data in relation to parents' sex, one item turned out to differ significantly between mothers and fathers' reports: fathers to a lesser extent reported that they had received the information that their child's illness was incurable (*p* = 0.048). When controlling for marital status, no significant differences were found. When analysing parents who were employed versus those who were not (due to studies, parental leave, unemployment, sick leave or being a housewife/‐husband), a significant difference was shown: those who were not employed to a lesser extent reported that their child got diagnostic information about their cancer compared with parents who were employed (*p* = 0.023).

## 4 DISCUSSION

In this study parents reported that not all children received diagnostic information. Almost all (93%) parents want information when their child's illness become incurable. In addition to this they reported that more than half of the children never received such information. The study also revealed that fathers to a lesser extent than mothers reported that they were informed that the child's illness was incurable.

The finding that parents reported their child being uninformed about the diagnosis and the severity of their illness is in line with findings in a Swedish registry study,^17^which concluded that less than half of the children (4–17 years) got information from their physician about the incurability of their cancer. Diagnosis and prognosis might have been communicated, but not registered.^17^ In our study, the children of the parents who reported that their child was uninformed about the diagnosis were quite young. However, it is unclear why so many children may not have received information, since evidence shows that even young children are able to understand and communicate their own perceptions of suffering from cancer.^18^ Again, it is important to consider that both diagnosis and prognosis may have been communicated by healthcare professionals several times, yet were not perceived as communicated by parents, possibly due to the extremely stressful situation. In a recent scoping review regarding communicating bad news in childhood cancer care, it was found that parents were sometimes so troubled and upset that this decreased their ability to listen to and understand the information delivered by physicians.^19^ Healthcare professionals may therefore need to regularly assess how well‐informed the child and parents are and repeat important information if needed.

The finding that the number of children attending the communication sessions decreased with increasing degree of severity of their illness is of interest, since according to the Convention on the Rights of the Child children are considered to have the right to know about their illness and prognosis. However, it is important to consider each child's situation and preferences. Some children might not want to know about diagnosis and prognosis,^20^ and some want truthful information but in a positive way leaving room for hope.^21^ When delivered, information should of course be in an age‐ and developmentally appropriate way.^22^ Research shows that parents are often considered to be favourable carriers of such information to their child.^23^ However, it has been found that parents found it very burdensome to be the messenger and to communicate the illness to their child with cancer.^24^ Results from previous research also showed that parents experienced major challenges and suffered from emotional distress when conveying information about the illness and they reported that they sometimes toned down the information and tried to be positive to protect their child.^24^ In the same study, parents shared their positive experiences of receiving facilitating support for their difficult task, including ‘preparation’, ‘books and resources’, ‘team engagement’ and ‘play’.^24^ However, parents cannot be expected to take on the full responsibility for communicating about illness and prognosis to their child, and any communication should aim to achieve a caring collaboration between healthcare professionals and parents, to ensure a family‐focused dialogue.

We claim that healthcare professionals always are primarily responsible for prognostically communication. However, it is important to keep in mind that healthcare professionals are facing challenging situations not only when conveying bad news^25^, ^26^ but also when being prevented by parents from disclosing the truth. Parents' reasons for this have been described as fear that the information will overwhelm the child,^27^ and that disclosure would negatively affect interactions with siblings and other relatives, leading to instability in the family.^22^ Further reasons for non‐disclosure were expected negative psychological consequences for the child such as reduced hope, seclusion, anxiety, and sadness.^28^

Being prevented from telling the child the truth is described as an ethical issue by healthcare professionals caring for children with cancer^7^ in their role as information providers to families. Healthcare professionals' knowledge about what is at stake for the parents related to disclosure versus non‐disclosure would most probably facilitate decision‐making and handling of the ethical issues.^29^ In a recent paper Rost and Mihailov (2021) argue against non‐disclosure, even though it is the wish of the parents.^27^ The authors state that disclosure is recommended since it will lead to continuing stability of the family, contrary to what parents fear.^27^ Moreover, research has shown that children might suffer from increased fear, anxiety, depression and social problems if not informed about their illness and prognosis.^28^ Research has also revealed that delayed disclosure to children might contribute to children's feelings of anger and betrayal.^28^ Yet, it should be noticed that close to all parents (93%) in our study want to receive information themselves when their child's illness become incurable.

Conversations on diagnostic and prognostic information should always include the triad of ‘stakeholders’: the child, the parents and the healthcare professionals. To ensure that optimal communication is attained, healthcare professionals need to assess children's and/or parents' communication preferences.^30^ In summary, there may be several explanations for parents' perceptions that their children were uninformed about diagnosis and prognosis and that the child did not attend the communication sessions, which all deserve attention in order to improve quality of care. We believe it is important to embrace the complexity of these difficult situations and acknowledge the depth of severity.

In this study, fathers to a lesser extent than mothers reported that they had received the information that their child's illness was incurable. Lannen and colleagues (2010) investigated how mothers and fathers grasp information about their child's illness and prognosis; their results also indicated differences between sexes, although the differences were not statistically significant.^4^ In the study by Lannen et al. (2010), it was shown that parents who did not suffer from previous depression, who had someone to share their experiences with and who considered that the communication about the child's incurability was delivered in a good and respectful way, were more likely to have grasped what was communicated.^4^ One possible reason for the significant difference between mothers and fathers in our cohort may be that fathers lacked one or several of these factors, although determining which factor might require further exploration, preferably through qualitative interviews.

As a final remark: we did not see any clear associations explaining the results showing why unemployed parents were less likely to report that their child got the information that he/she had cancer as compared with employed parents. A possible explanation may be that unemployment may lead to other burdening worries that impact the parental role.^31^

### 4.1 Discussion on methods

The strengths of this study include its design and data collection methodology. By using a questionnaire, a large population could be reached, anonymity was enabled, and interviewer bias was avoided. Other strengths of this study were the nearly equal sex distribution and the relatively small number of missing responses.

### 4.2 Study limitations

A debatable limitation is recall bias: the data are self‐reported perceptions of events that happened in the past and strong emotions may possibly blur memories. Because of cultural differences in grief and bereavement care in other countries and cultures and since the results of this study are based on a Swedish nationwide population, we cannot generalise our findings beyond cancer‐bereaved parents in Sweden.

## 5 CLINICAL IMPLICATIONS

In this study we found that more than half of the children had not received the information about their illness being incurable. Breaking bad news has been found very challenging by health care professionals. However, there are supportive tools at hand, one of them is SPIKES, a standardised protocol for communicating bad news. The tool is meant as a strategy more than a script.^32^ Other approaches to support healthcare professionals improve communication, specifically focussing on children and their family include: to first listen and be sensitive to cues from the child, to talk with a language that is clear and as simple as possible and developmentally appropriate, to make sure to have adequate time so the communication can be paced, to encourage questions and ask the child to repeat back what has been said, in order to make sure that they have understood, to offer children books and literature with content fitting the situation at hand and lastly, to pay attention to if the child is open for using creative activities such as painting, drawing and storytelling to help to facilitate discussions.^28^, ^33^

Even though close to all parents want information when their child's illness become incurable, not all parents want to reveal this to their children. Recommendations, of helpful actions for disclosure of a bad prognosis to children when parents refuse, include to observe interaction and patterns of communication in the family to tailor prognostic disclosure to each family, to communicate the valuable outcomes of prognostic disclosure, to promote a feeling of cultural safety by acknowledging the values and beliefs of the family and to consult the ethics committee if needed.^27^

Families may have various resources and needs, and some children and parents may need repeated conversations to grasp what has been said and time to process all the information they received, or to ask questions. It is also important to be aware that mothers' and fathers' perceptions of what is clear sometimes differ, and that family‐focused care includes taking the needs of all family members into account. Therefore, a possible next step is to assess preferences for information and communication for all family members early in the illness process.

## 6 CONCLUSIONS

A vast majority of both mothers and fathers would like to know themselves when their child's illness becomes incurable. However, more than half of bereaved mothers and fathers stated that challenging information, that the cancer was incurable, was not conveyed to their child. Healthcare professionals should strive for ensuring that communication involves the triad of stakeholders: child, parents, and healthcare professionals. However, it seems a challenging task for healthcare professionals to communicate and at the same time take all family members best interest into account.

## CONFLICT OF INTEREST

The authors have no conflicts of interest to declare.

## Data Availability

The data that support the findings of this study are available from the corresponding author upon reasonable request. Study trial ethical registration number (2015/2183‐31/5).
